# Dosimetric Comparison of Upfront Boosting With Stereotactic Radiosurgery Versus Intraoperative Radiotherapy for Glioblastoma

**DOI:** 10.3389/fonc.2021.759873

**Published:** 2021-10-28

**Authors:** Gustavo R. Sarria, Zuzanna Smalec, Thomas Muedder, Jasmin A. Holz, Davide Scafa, David Koch, Stephan Garbe, Matthias Schneider, Motaz Hamed, Hartmut Vatter, Ulrich Herrlinger, Frank A. Giordano, Leonard Christopher Schmeel

**Affiliations:** ^1^ Department of Radiation Oncology, University Hospital Bonn, Bonn, Germany; ^2^ Department of Neurosurgery, University Hospital Bonn, Bonn, Germany; ^3^ Department of Neurology, Division of Neuro-Oncology, University Hospital Bonn, Bonn, Germany

**Keywords:** dose escalation, SRS, IORT, kilovoltage, glioblastoma

## Abstract

**Purpose:**

To simulate and analyze the dosimetric differences of intraoperative radiotherapy (IORT) or pre-operative single-fraction stereotactic radiosurgery (SRS) in addition to post-operative external beam radiotherapy (EBRT) in Glioblastoma (GB).

**Methods:**

Imaging series of previously treated patients with adjuvant radiochemotherapy were analyzed. For SRS target definition, pre-operative MRIs were co-registered to planning CT scans and a pre-operative T1-weighted gross target volume (GTV) plus a 2-mm planning target volume (PTV) were created. For IORT, a modified (m)GTV was expanded from the pre-operative volume, in order to mimic a round cavity as during IORT. Dose prescription was 20 Gy, homogeneously planned for SRS and calculated at the surface for IORT, to cover 99% and 90% of the volumes, respectively. For tumors > 2cm in maximum diameter, a 15 Gy dose was prescribed. Plan assessment was performed after calculating the 2-Gy equivalent doses (EQD2) for both boost modalities and including them into the EBRT plan. Main points of interest encompass differences in target coverage, brain volume receiving 12 Gy or more (V_12_), and doses to various organs-at-risk (OARs).

**Results:**

Seventeen pre-delivered treatment plans were included in the study. The mean GTV was 21.72 cm^3^ (SD ± 19.36) and mGTV 29.64 cm^3^ (SD ± 25.64). The mean EBRT and SRS PTV were 254.09 (SD ± 80.0) and 36.20 cm^3^ (SD ± 31.48), respectively. Eight SRS plans were calculated to 15 Gy according to larger tumor sizes, while all IORT plans to 20 Gy. The mean EBRT D_95_ was 97.13% (SD ± 3.48) the SRS D_99_ 99.91% (SD ± 0.35) and IORT D_90_ 83.59% (SD ± 3.55). Accounting for only-boost approaches, the brain V_12_ was 49.68 cm^3^ (SD ± 26.70) and 16.94 cm^3^ (SD ± 13.33) (p<0.001) for SRS and IORT, respectively. After adding EBRT results respectively to SRS and IORT doses, significant lower doses were found in the latter for mean D_max_ of chiasma (p=0.01), left optic nerve (p=0.023), right (p=0.008) and left retina (p<0.001). No significant differences were obtained for brainstem and cochleae.

**Conclusion:**

Dose escalation for Glioblastoma using IORT results in lower OAR exposure as conventional SRS.

## Introduction

Since the standardization of adjuvant chemo-radiotherapy (CRT) for Glioblastoma (GB) over 15 years ago (1, 2), scarce progress has been achieved in order to improve the control and survival outcomes of these patients. Local recurrence, within the resection cavity or its close surroundings, remains to be the most frequent pattern of failure after combined treatment, including those patients in whom complete resection can be achieved, which promptly leads to detrimental clinical evolution and impaired quality of life (3, 4).

Under this rationale, different approaches have been proposed including new systemic agents and different RT modalities; however, they have repeatedly failed to improve the expected control and survival profiles (5, 6). Early strategies to improve such outcomes encompass dose-escalated RT. Different techniques have been assessed along the past four decades pre-dating the Temozolomide era, with mixed results. Increasing normofractionated doses in different levels was tested in the phase I RTOG 9803 study, meeting a safety profile up to 84 Gy and suggesting an improvement in survival outcomes at this dose level (7). Nevertheless, further studies have failed to confirm this hypothesis (8). Stereotactic radiotherapy, in either single (SRS) or multiple fractions (FSRT), has been investigated as well. Two major trials assessing both strategies (RTOG 9305 and RTOG 0023) in postoperative residual tumors could not demonstrate a significant survival benefit, although patients who achieved a complete resection allegedly profited from FSRT (9, 10). The main limitation of this strategy lies on toxicity, as increased rates of radionecrosis (RN) have been observed and are to be expected due to the usually large irradiation volumes.

Employing both low-dose (LDR) and high-dose rate (HDR) brachytherapy as an upscaling method has been additionally evaluated since the late 1980’s, with substantial differences between prescription doses, toxicity and control outcomes in reporting (11). Due to major concerns regarding radioprotection and an apparent increased rate of RN, probably related to a deeper dose prescription in healthy tissue, utilizing this approach has declined over time. Despite these issues, promising control rates have been published (12, 13). Similarly, earlier reports of electron-based intraoperative radiotherapy (IORT) suggested inspiring outcomes regarding local control, although no major impact on survival was noted.

Lately, based on these previous publications, the first clinical experiences of IORT with low-energy x-rays (kilovoltage) have described preliminary encouraging results in terms of both disease control and toxicity rates (14, 15). A most relevant feature of kilovoltage-IORT is a steep fall-off dose beyond the applicator, which allows preserving the surrounding unaffected tissue with an approximated 30% of the isodose reaching 1 cm (16).

This study provides a dosimetric comparison of pre-operative SRS against IORT as dose-escalation approaches in addition to normofractionated external-beam radiotherapy (EBRT) for patients diagnosed with glioblastoma.

## Methods

### Patients and Procedures

**Figure 1:**
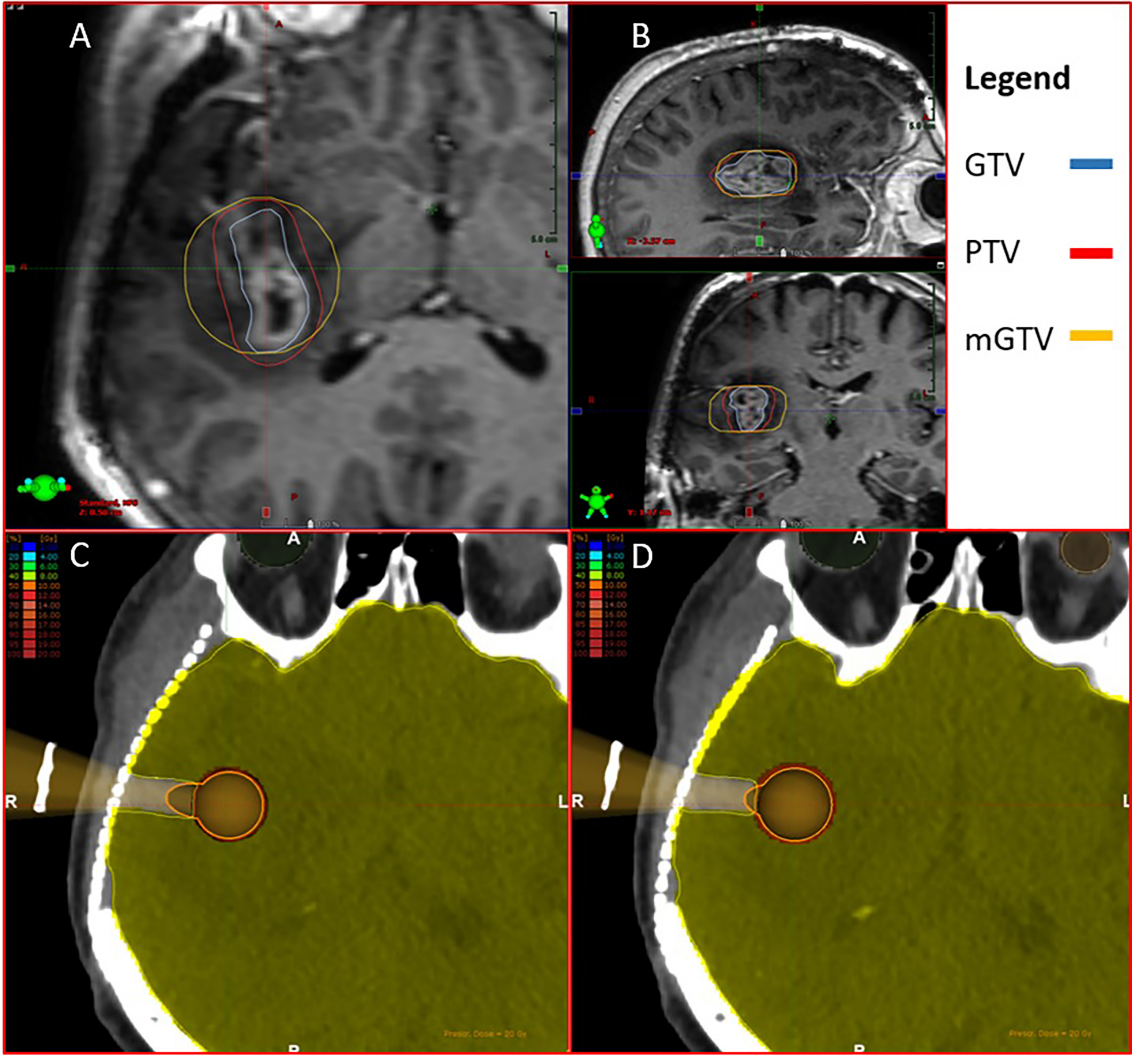
**(A, B)** Generated contours for intraoperative radiotherapy (IORT) and stereotactic radiosurgery (SRS) boosting. GTV: gross target volume, PTV: planning target volume, mGTV: modified gross target volume. mGTV was adjusted to fit the immediate larger apposite applicator. **(C, D)** Simulated path for the applicator placement. The healthy brain contour was removed from the hypothetical surgical trajectory.

Patients diagnosed with GB and treated according to the standard of care (2) were retrospectively screened, in order to retrieve their imaging series. Simulation-CTs and pre- and post-operative MRIs were identified and rigidly co-registered. Those patients having tumors closer than 1 cm to either chiasma, optical nerves or brainstem were excluded. Standard 60-Gy EBRT volumes were delineated including a T1- and FLAIR-weighted postoperative MRI on each patient’s set, accounting for any residual gross tumor (GTV) and the resection cavity, a 1.5-cm CTV modified to encompass any surrounding edema, and 0.3-cm PTV. To simulate an upfront pre-operative SRS boost, the preoperative GTV was reconstructed in all cases based on the MRI T1-weighted tumoral uptake. For defining the SRS planning target volume (PTV), a 2-mm isotropic expansion from the GTV was applied. For IORT, a modified (m)GTV was expanded from the pre-operative MRI-T1 GTV according to the outermost borders of the lesion, in order to mimic a round cavity as during IORT with a spherical applicator. Furthermore, mGTV contours were enlarged circumferentially and homogeneously to fit the immediate larger applicator. Both GTV and mGTV volumes were subtracted from normal brain tissue delineations. An exemplary case is displayed in **Figures 1A, B**.

The 60-Gy plans were set to cover 95% of the volume with 100% of the intended dose. Dose prescription was 20 Gy, homogeneously planned for SRS and prescribed to the surface of the IORT applicator, to cover 99% and 90% of the volumes, respectively. For SRS plans with GTVs larger than 2 cm in maximum diameter, a 15 Gy dose was prescribed. All contours, EBRT and SRS calculations were performed on Eclipse 13.6 for TrueBeam STx (Varian Medical Systems, Palo Alto, CA, USA), equipped with a High Definition Multileaf Collimator (HD 120MLC). Intraoperative RT contours were exported to and planned with Radiance (GMV SA, Madrid, Spain), employing a Monte Carlo simulation algorithm for low-energy x-rays delivered with Intrabeam (Carl Zeiss Meditec AG, Jena, Germany).

Delivered SRS and IORT doses to OARs were converted to 2-Gy equivalents (EQD_2_) considering a 2-Gy α/β factor (17) if exceeding a 2-Gy exposure in this single application. These dose results were added to the 60-Gy EBRT plans for final assessment.

Constraints were adopted from the INTRAGO II protocol (NCT02685605) for both boost modalities with a maximum 8-Gy tolerance dose to OARs, encompassing the brain volume (cm^3^) receiving 12 Gy (V_12_) to be lower than 10 cm^3^. In addition, the combined 60-Gy EBRT and boost doses were set to a total EQD_2_ D_max_ 66 Gy for brainstem, D_max_ 55 Gy for chiasma and optical nerves, cochleae D_mean_ 35 Gy and retinae D_mean_ 45 Gy.

### Endpoints

Main points of interest include differences in target coverage (V_99_ = 99% for SRS and V_90_ = 100% for IORT), brain V_12_ between only SRS and IORT plans, constraint compliance, and maximum (D_max_ = 0.03 cm^3^) and mean dose (D_mean_) exposure to brainstem, chiasma, optic nerves, retinae and cochleae, correspondingly.

### Statistical Analysis

Comparative mean and median measurements and their corresponding standard deviation (SD) or ranges are described accordingly. The *t*-test was employed to determine the statistical significance between differences in continuous variables, assuming a p ≤ 0.05.

### Ethics

This investigation was released from Institutional Review Board (IRB) approval due to its retrospective comparative planning nature. All data sets were anonymized prior to the analysis and no personal information is consigned in this manuscript, in concordance with the principles of the Declaration of Helsinki

## Results

### Patients and Planning Features

After patient selection, seventeen pre-delivered treatment plans were included in the study. The mean preoperative GTV volume was 21.72 cm^3^ (SD ± 19.36) and mGTV 29.64 cm^3^ (SD ± 25.64). The mean EBRT and SRS PTVs were 254.09 (SD ± 80.0) and 36.20 cm^3^ (SD ± 31.48), respectively. Eight SRS plans were calculated to 15 Gy according to larger tumor sizes, whereas all IORT plans to 20 Gy. The median applicator size was 35 mm (15 – 50). These features are summarized in **Table 1**.

**Table 1:** Planning baseline features.

Structure volume (cm^3^)		
	Mean	SD
GTV	21.72	19.36
PTV	36.2	31.48
mGTV	29.64	25.64
EBRT	254.09	80
Boost delivered doses (Gy)		
	20	15
SRS (%)	52.94	47.06
IORT (%)	100	
Applicator diameter (mm)		
	Median	Range
Applicator size	35	15 - 50

GTV, gross target volume; PTV, planning target volume; mGTV, modified gross target volume; SRS, stereotactic radiosurgery; IORT, intraoperative radiotherapy.

The mean EBRT D_95_ was 97.13% (SD ± 3.48), the SRS D_99_ 99.91% (SD ± 0.35) and IORT D_90_ 83.59% (SD ± 3.55). The mean SRS conformity index (CI) was 1.26 (SD ± 0.35). Accounting for only-boost approaches, the brain V_12_ was 49.68 cm^3^ (SD ± 26.70) and 16.94 cm^3^ (SD ± 13.33) (p<0.001) for SRS and IORT, respectively. No SRS patients in contrast to eight IORT patients could fulfill the V_12_ tolerance criterion. All IORT plans with mGTVs under 30 cm^3^ or applicators smaller than 3.5 cm and a plan with a 44.8 cm^3^ lesion and a 45 mm applicator achieved V_12_ exposures under 10 cm^3^. Two additional IORT patients showed V_12_ exposures of 13.16 and 12.84 cm^3^, with smaller target volumes as the abovementioned. Regarding the other OAR constraints, four SRS patients reached doses over the preset tolerance of brainstem, chiasma, left and right optic nerves, in comparison to three IORT patients with overdosing on the three latter structures.

After SRS and IORT EQD_2_ calculations and adding EBRT results, mean brainstem D_max_ was 44.06 Gy (SD ± 17.75) and 42.91 (SD ± 16.84; p=0.228), respectively. Combined SRS and IORT exposure to chiasma was D_max_ 28.96 Gy (SD ± 19.09) and 27.72 Gy (SD ±18.50; p=0.01), right optic nerve D_max_ 19.51 Gy (SD ± 18.69) and 19.48 (SD ± 19.48; p=0.977), and left optic nerve D_max_ 15.71 Gy (SD ± 16.23) and 14.60 Gy (SD ± 14.66; p=0.023). For mean D_mean_ exposures, right retina received 7.28 Gy (SD ± 6.37) and 6.50 Gy (SD ±5.71; p=0.008), left retina 6.24 Gy (SD ± 5.61) and 5.66 Gy (SD ± 5.57, p<0.001), right cochlea 10.74 Gy (SD ± 10.74) and 11.85 Gy (SD ± 15.35; p=0.295), and left cochlea 9.43 Gy (SD ± 10.75) and 9.72 Gy (SD ± 10.96; p=0.645). These results are shown in **Table 2**.

**Table 2:** Organs at risk dosimetric differences.

Dose exposure					
Organs at risk	EBRT+SRS		EBRT+IORT		p
	Mean	SD	Mean	SD	
Brain V12 (no EBRT)	49.68	26.7	16.94	13.33	**<0.001**
Brainstem Dmax	44.06	17.75	42.91	16.84	0.228
Chiasma Dmax	28.96	19.09	27.72	18.5	**0.01**
Left optic nerve Dmax	15.71	16.23	14.6	14.66	0.023
Right optic nerve Dmax	19.51	18.69	19.48	19.48	0.977
Left retina Dmean	6.24	5.66	5.61	5.57	**<0.001**
Right retina Dmean	7.28	6.37	6.5	5.71	**0.008**
Left cochlea Dmean	9.43	10.75	9.72	10.96	0.645
Right cochlea Dmean	10.74	14.9	11.85	15.35	0.295

EBRT, external beam radiotherapy; SRS, stereotactic radiosurgery; IORT, intraoperative radiotherapy; V_12_, volume receiving 12 Gy. The brain V_12_ was not added to EBRT doses due to practical reasons. Statistically significant dosimetric differences are displayed and highlighted.

**Figure 2:**
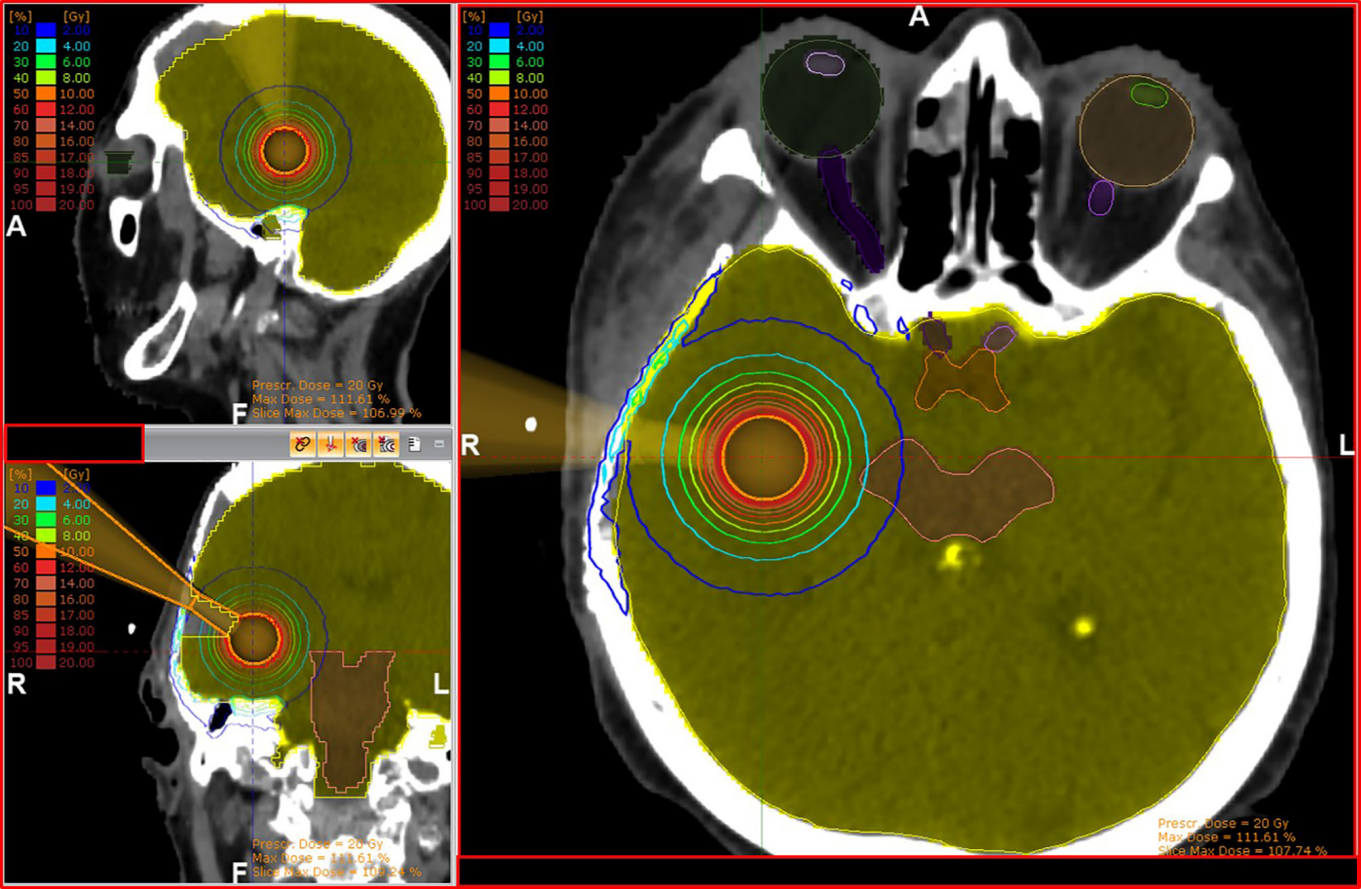
Exemplary case with three-dimensional views of isodose line distribution for a 2.5-cm IORT applicator. Doses reaching the reconstruction plaque should be disregarded to resemble a real-world surgical situation.

**Figure 3:**
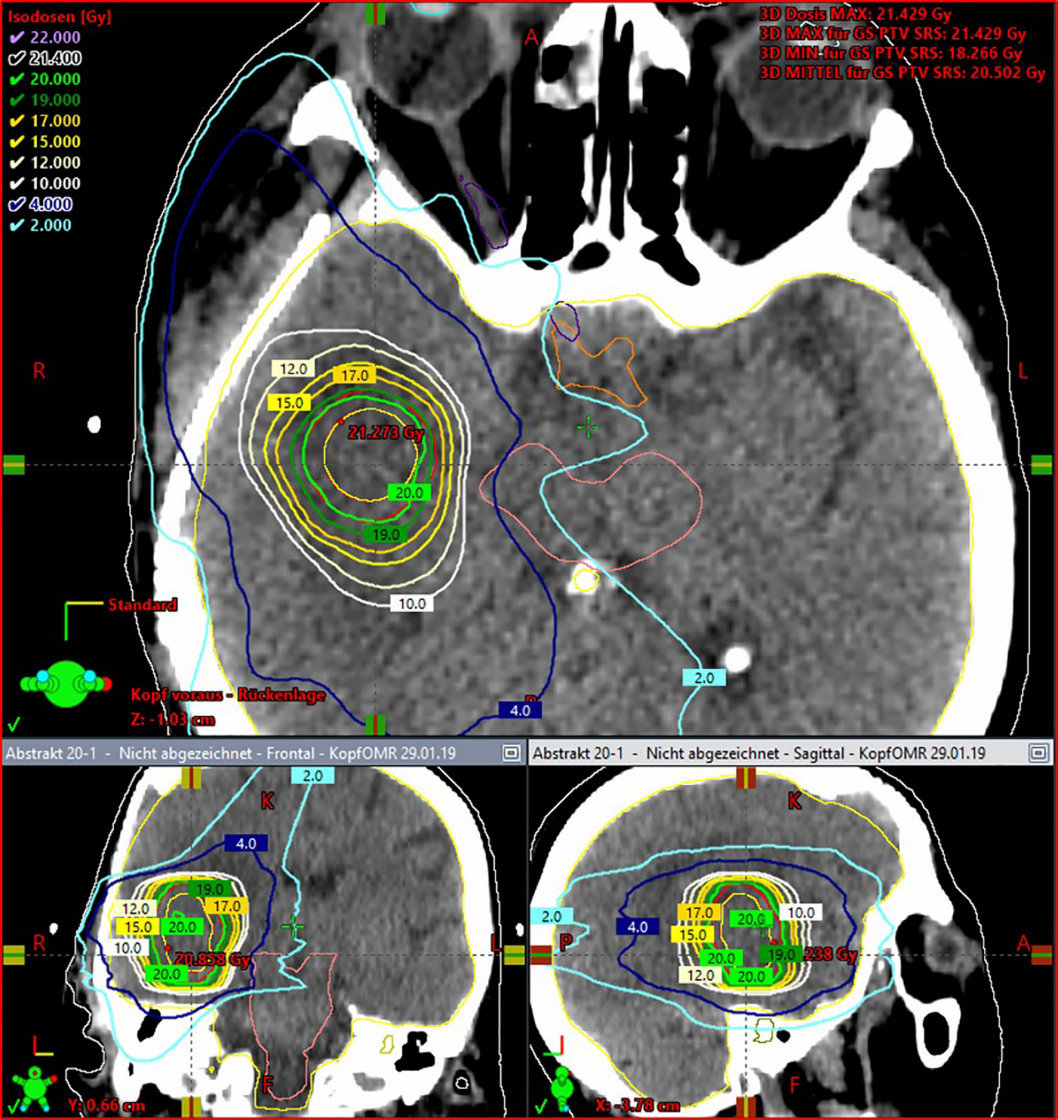
Exemplary case with three-dimensional views of isodose line distribution for upfront SRS boosting, homogeneously delivered to a 2-mm PTV.

Graphic examples of tissue exposure and dose distribution can be observed in **Figures 2** and **3**. The detailed resulting doses for the entire cohort are displayed in **Appendix 1**.

## Discussion

This is the first dosimetric comparison of pre-operative SRS and kilovoltage IORT as dose-escalation approaches for patients with GB. The rationale for comparing these modalities lies on both the ongoing INTRAGO II trial and historical results from randomized trials, which failed to demonstrate clinical benefit of post-operative SRS (10). Furthermore, an inherent feature of post-surgical irradiation is a significant shape variability of resection cavities (18), which usually leads to rather large boost volumes and increased rates of toxicity. Despite some minor shortcomings, reconstructing the pre-operative GTVs yields a more reliable and homogeneous comparison mean between upfront SRS and IORT deliveries. Nevertheless, employing dose-escalated RT is nowadays a controversial strategy to improve GB treatment outcomes and must be explored in a prospective manner. Previous experiences from the RTOG 9305 and 0023 failed to demonstrate any survival benefit when targeting residual disease (9, 10). On the contrary, although not powered for this endpoint, the phase I RTOG 9803 trial suggested a survival benefit for these patients in comparison to the standard of care (7). This hypothesis has been further pondered according to mathematical models suggesting enhanced results after dose-escalation, based on both clinical and *in-vitro* data (19, 20). From the clinical perspective, a relevant feature inherent to the previously mentioned studies was assessing patients with residual tumors and targeting these lesions, which does not allow a comprehensive management of the entire surgical cavity. This should be taken into consideration for dose-escalation strategies, as the prognostic value of residual disease is widely known to be impairing in terms of survival. The ongoing INTRAGO II trial (NCT02685605) will help elucidate this matter.

Target volume coverage was assessed according to institutional and international EBRT (D_95_ = 100%), SRS (D_99_ = 99%) and brachytherapy (D_90_ = 100%) planning standards (21–23). A major discrepancy was observed between both boost techniques in terms of target coverage. The mGTV portion lying behind the “neck” of the applicator does not receive a significant dose, implying a poor distribution at this level. However, in a real-life setting, this area represents the surgical entry path and as such, it would not be accounted for calculations. In addition, the contours generated on this platform cannot be modified to allow hollow structures. Therefore, these dosimetric differences, although reported, must be disregarded due to practical reasons. A direct or statistical comparison would not be feasible according to the abovementioned and it is suggested to assess each single modality by separate. A graphical example can be seen in **Figures 1C, D**.

Prescription doses, selected in two levels, resemble of those according to the RTOG 9305 protocol for the larger lesions and the INTRAGO II protocol for the smaller ones. The latter allows a therapeutic range between 30 to 20 Gy, according to the applicator’s proximity to OARs (< 1.5 cm) or exposure over tolerance (D_max_ 8 Gy). In this cohort, all IORT plans were calculated to 20 Gy delivered at the surface, easing the comparison between both techniques and mimicking real-life conditions, mostly for SRS plans. Due to these handicaps and difficulties in achieving the predefined constraints, eight SRS plans had to be performed with a 15-Gy prescription, in contrast to no IORT plans. Despite these adjustments and after adding EBRT doses, four SRS plans could not meet the non-V_12_ constraints in comparison to three IORT plans. Nevertheless, it is noteworthy that, although significant differences between some mean OAR doses were observed, these outcomes might not be clinically significant given the marginal numeric variability. This is naturally variable amongst patients according to the irradiated area or volume, although clear advantage of IORT over SRS can be anticipated for smaller irradiated targets, thus increased healthy tissue sparing. Doses reaching the unaffected organ can be restricted due to its steep fall-off profile and no need of any security margin around the mGTV (resection cavity). This is the most relevant factor for significantly diminishing brain V_12_ with this approach. It is worth to mention that the SRS PTV volumes herein obtained were considerably larger than those of mGTV for IORT and this might be highly variable according to local standards. However, even when considering applying smaller or no PTV expansions, the dose-distribution profile of kilovoltage still results superior in comparison to megavoltage. The 2-mm PTV expansion used in this study represents our local standard for linear accelerator-based SRS, which might vary according to local standards or available technology, such as multi-source cobalt-based SRS. Regarding the planning technique, all SRS plans were calculated homogeneously, as described under Methods. This was decided according to our local standards, which in our experience yields similar surrounding tissue exposure, as different other features influence the fall-off dose profile, such as number of rotations, arcs, amongst others. In the setting of a pre-operative SRS, as proposed in this study, intratumoral dose-escalation would not play a relevant role, when the cavity border is of higher importance.

It is worth highlighting the potential advantage of lower V_12_ results as a RN prediction tool. With almost all previously reported experiences converging on a similar point, it is of considerable relevance to maintain this parameter within acceptable ranges (17, 24). In our cohort, patients who had mGTV smaller than 30 cm^3^ or were planned with ≤ 3.5 cm applicators yielded improved V_12_ results. On the contrary, no SRS plan would be acceptable for clinical practice, even after limiting the prescription doses to 15 Gy. It should be considered as well, that no specific constraints for healthy brain irradiation have been defined when combining IORT/SRS to EBRT. Therefore, increased attention should be given to exposed unaffected tissue. Based on published clinical experiences, an estimated 15 – 20% grade 3 RN is to be expected after combining EBRT and IORT; however, no variables were individually assessed to determine which of them could mostly contribute to these events (14, 15). These outcomes are closely bound to earlier dosimetric data, which described a direct relationship between larger applicator sizes and increased doses in depth (16). According to this information, patient selection must be carefully performed in order to avoid undesired adverse events.

Limitations to this investigation include both its retrospective nature and definition of intraoperative target volumes. Although this method might be the most accurate to retrospectively simulate a surgical bed, intrinsic anatomical variations inherent to the surgical procedure and tissue re-accommodation, which currently cannot be accounted for, are still to be present. In addition, due to the differences in dose levels, a plan sum for both EBRT and SRS/IORT modalities seems impractical when assessing the healthy brain exposure. Implications of this drawback might include an inability to calculate accurately the actual clinical risks of dose escalation. Parameters such as V_12_ have been studied largely for single fraction applications, with no available data on combined normo- and ablative fractionations. Results of ongoing clinical trials will help to overcome this shortcoming, based on their already available preliminary results (14, 15). Furthermore, the relative biological effectiveness (RBE) factor of kilovoltage therapy has not been considered amongst the resulting calculations of IORT (25). The large doses applied and low reliability of the linear-quadratic model at such levels, in addition to a lacking clinical validation, led us to omit its application. The linear-quadratic model itself carries certain flaws when assessing large doses per fraction; however, it has been widely adopted to allow comparisons between treatment schemes and pool data, even in settings like this (26). We suggest deeming all available factors when considering IORT-boosting. Taken together, this investigation provides relevant information regarding the exposed healthy brain tissue and OARs for both dose-escalation strategies. Intraoperative imaging with the applicator *in situ* is required to accurately reproduce treatment delivery.

## Conclusion

Dose escalation with IORT yields significant lower healthy brain V_12_ exposure in comparison to pre-operative SRS for Glioblastoma patients, while allowing a higher dose delivery to the surgical bed. Larger IORT applicator sizes might relate to increased V_12_ results. Careful patient selection must be performed in order to diminish RN rates.

## Data Availability Statement

The original contributions presented in the study are included in the article/**Supplementary Material**. Further inquiries can be directed to the corresponding author.

## Ethics Statement

This investigation was released from Institutional Review Board (IRB) approval due to its retrospective comparative planning nature. All data sets were anonymized prior to the analysis and no personal information is consigned in this manuscript, in concordance with the principles of the Declaration of Helsinki.

## Author Contributions

GS: study conceptualization and design, data production, collection and statistical analysis, manuscript drafting and editing. ZS: data production and collection. TM: study design, data production and analysis. JH: data production. DS: manuscript review and editing. DK: manuscript review and editing. SG: data review. MS: manuscript review. MH: manuscript review. HV: manuscript review. UH: manuscript review. FG: study design, manuscript review and editing. LS: study conceptualization and design, manuscript review and editing. All authors contributed to the article and approved the submitted version.

## Conflict of Interest

GS: personal fees from Carl Zeiss Meditec AG and personal fees from Roche Pharma AG, not related to this work. FG: research grants and travel expenses from ELEKTA AB; grants, stocks, travel expenses and honoraria from NOXXON Pharma AG; research grants, travel expenses and honoraria from Carl Zeiss Meditec AG; travel expenses and honoraria from Bristol-Myers Squibb, Roche Pharma AG, MSD Sharp and Dohme GmbH and AstraZeneca GmbH; non-financial support from Oncare GmbH and Opasca GmbH, not related to this work.

The remaining authors declare that the research was conducted in the absence of any commercial or financial relationships that could be construed as a potential conflict of interest.

## Publisher’s Note

All claims expressed in this article are solely those of the authors and do not necessarily represent those of their affiliated organizations, or those of the publisher, the editors and the reviewers. Any product that may be evaluated in this article, or claim that may be made by its manufacturer, is not guaranteed or endorsed by the publisher.
